# *In-situ* restoration of one-stage partial nitritation-anammox process deteriorated by nitrate build-up via elevated substrate levels

**DOI:** 10.1038/srep37500

**Published:** 2016-11-24

**Authors:** Xiaolong Wang, Dawen Gao

**Affiliations:** 1 State Key Laboratory of Urban Water Resource and Environment, Harbin Institute of Technology, Harbin 150090, China

## Abstract

The one-stage partial nitritation and anammox process (PN/A) has been a promising microbial process to remove ammonia from wastewater especially with low carbon/nitrogen ratio. The main breakdown was the deterioration caused by overgrowth of nitrite oxidizing bacteria (NOB) resulting effluent nitrate build-up in the PN/A process. This study presented an *in-situ* restoring strategy for suppressing NOB activity in a one-stage granular PN/A system deteriorated over 2 months, using elevated concentrations of substrates (ammonia and nitrite) under limited dissolved oxygen level. The results showed that the NOB activity was successfully suppressed after 56 days of restoration, and finally the ratio of produced nitrate/consumed ammonium was reduced from 36.8% to 7%. On day 66 the nitrogen removal rate obtained as 1.2 kg N/(m^3^·d). The high FA level (5–40 mg/L) and low dissolved oxygen (<0.13 mg/L) were responsible for NOB suppression. From quantitative PCR (qPCR) analysis, after this restoration, anammox bacteria had a widely growth, and AOB stay stable, but *Nitrospira* increase and *Nitrobacter* declined. High amount of NOB was still persistent in the granules, which was not easy to wash-out and threaten the deammonification performance.

The increasing demands of nitrogen discharge limit from wastewater with minimized energy consumption has always been a great challenge for wastewater treatment. As a novel nitrogen removal pathway discovered in early 1990s, anaerobic ammonium oxidation (anammox) is the process in which anammox bacteria use ammonium as electron donor, and nitrite as electron acceptor, to generate dinitrogen gas under anaerobic condition^1^. So anammox must be applied coupling with partial nitritation (PN/A) in different styles to treat ammonia wastewater with low carbon/nitrogen ratio in one-stage or two-stage^2^. The PN/A process is catalyzed by aerobic ammonia oxidizing bacteria (AOB) and anammox bacteria, respectively (as formula 1). Although two-stage PN/A process obtains higher efficiency, one-stage PN/A is considered as more economical^3^,^4^. Compared to traditional nitrification-denitrification treatment, one-stage PN/A is also a more cost-effective and energy efficient process^5^,^6^.


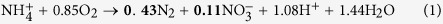


There are two main challenges to obtain a stable performance in PN/A process. One inherent challenge is the slow growth of anammox bacteria (doubling time of 8.3–11 days)^7^,^8^. So the long biomass retention is an basic demand to prevent biomass washout from reactor. One way is using biofilm or granule system^9^, like moving bed reactor^10^,^11^ or fixed bed reactor^12^. In suspended growth system, external settling time or device is also possible to prevent biomass loss in the outflow^13^. Because AOB has shorter doubling time (7–8 h) than anammox bacteria, AOB is able to be retained along with anammox bacteria^14^,^15^,^16^. The other main obstacle is the stable performance of partial nitritation, in other words, is the enriching of AOB and meanwhile suppressing nitrite oxidizing bacteria (NOB). It was evident that the limit step in PN/A process was the partial nitritation^17^,^18^. Although with similar ecology habits, these two kinds of bacteria can be distinguished by slightly different growth rates under particular environment conditions. It is almost consensus that high temperature (over 25 °C)^19^, low dissolved oxygen (DO, below about 1–2 mg/L)^20^,^21^, intermittent aeration with interval above 15 min^22^, all benefit to enrich AOB. The inhibitors such as free ammonia (FA) and free nitrous acid (FNA) also have advantages on suppress NOB for its low toleration^23^.

To date, at least 100 full-scale PN/A installations have been operated worldwide^24^. Even so, out of control of NOB resulting nitrate build-up in the effluent remained one of the major issues in the full-scale installations^25^. Some full-scale PN/A system faced the problem of above 30% of nitrate build-up in effluent^26^. Considering the scarcity of massive anammox biomass for reinoculation, restoration study for the deteriorated PN/A processes is needed. Some inhibitors had been tested in the *in-situ* restoration of nitrate build-up in one-stage PN/A process, such as hydrazine, hydroxylamine, organic matter, ultrasonic treatment or aeration patterns. Hydrazine is the intermedium of anammox process. Long-term addition of 4 mg/L hydrazine had strong inhibition effect on NOB, meanwhile, the effect of promoting anammox bacteria growth and partially inhibiting AOB also improved the nitrogen removal efficiency of PN/A process^27^. Hydroxylamine is also an intermedium of AOB and anammox bacteria, so it can promote both of them. Although the single suppressing of 20 mg/L hydroxylamine on NOB was reversible, combining with SRT control of 40 days, NOB was successfully suppressed in one-stage PN/A process^28^. Organic matter could suppress NOB growth and promote anammox growth at the same time, and to same certain extent, reduce effluent nitrate production by introduction of denitrification^29^,^30^. It was also reported 0.09 kJ/mg VSS of ultrasonic treatment benefited to enhance the AOB and depress NOB activity^31^. A certain anoxic period was able to slow down NOB metabolism, so intermittent aeration with long enough anoxic period (>15–20 min) and aerobic periods (not exceed the specific lag phase of NOB) was sufficient for suppressing NOB, which had been applied in full-scale plant^22^,^32^,^33^.

The purpose of this study was to restore a deteriorated lab-scale granular PN/A system suffered nitrate build-up by inhibiting NOB activity under gradually elevated concentration of ammonia and nitrite under limited DO level. The restoration stage was evaluated by stoichiometry ratios of typical PN/A process, and the main inhibition factors associated was discussed. The 16S copy numbers of functional microorganism (anammox bacteria, AOB and NOB) in the restoration stages were also determined. At last, the possibility to treat the NOB overgrowth in deterioration PN/A system was concluded.

## Result

### Performance in restoration stages

**Figure 1:**
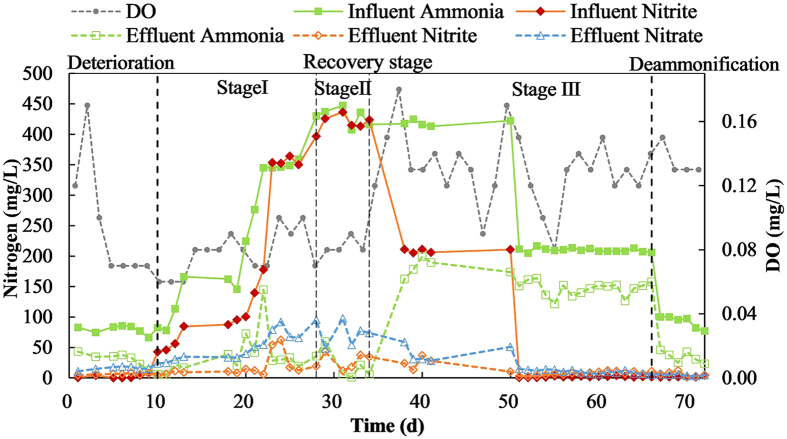
The performance of nitrogen removal in restoration process.

**Figure 2:**
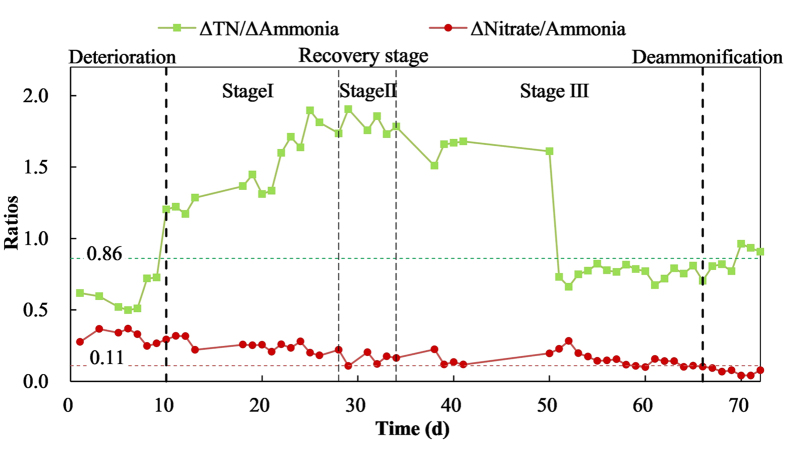
The ratio variations of one-stage PN/A system in restoration stages. Value 0.11 and 0.86 were the theoretical stoichiometric ratios of ΔNitrate/ΔAmmonia and ΔTN/ΔAmmonia, respectively.

The one-stage granular PN/A reactor was operated at 32 °C, 7.7 of pH, DO lower than 1.3 mg/L with intermitted aeration, and was fed with influent with 84 mg/L NH_4_^+^-N at the beginning. At the beginning of the experiment, the PN/A system suffered high nitrate production for at last two months, with the initial loading rate was about 2 kg N/(m^3^·d). The performance of different stages of deterioration, restoration and deammonification in terms of ammonium, nitrite and nitrate is shown in Fig. 1. From the performance of deterioration before the restoration process, about 17 mg/L nitrate in effluent (>30% of the removed ammonia) caused the two ratios of ΔTN/ΔAmmonia and ΔNitrate/ΔAmmonia deviated from theoretical value (Fig. 2).

The restoration process (day 10–66) contained three stages as variations of substrate concentrations (Fig. 1). From day 10–28, the system was feed with substances with gradually increasing concentrations. Starting from day 10, nitrite was introduced to this system to expect to improve the deammonification performance. The concentration of NO_2_^−^-N was gradually increased according to its removal efficiency in effluent (>95%). At the same time, doubled the concentration of NH_4_^+^-N to maintain external ammonium for deammonification simultaneously. Considering above 400 mg/L of influent nitrite and ammonia nitrogen was a danger level to anammox bacteria from operation experiences in our lab, ammonium concentration began to synchronize to that of nitrite from day 23–33. From day 28–34 the system entered the stable stage with the highest substrate concentrations. The concentrations of NO_2_^−^-N and NH_4_^+^-N were stabilized at around 420 mg/L for one week. High substance concentrations were expected to stable the suppressing effect on NOB. From day 34–66, the substance concentrations were falling down. In this period, the introduced nitrite would be removed and this system was transformed to run as the way of deammonification gradually. At day 34, NO_2_^−^-N concentration halved with unchanged NH_4_^+^-N concentration, and then after one week, no NO_2_^−^-N supplied and NH_4_^+^-N concentration halved. The system obtained the volumetric loading of nitrogen removal of 1.2 kg N/(m^3^·d) at last.

### Variations of stoichiometric ratios in PN/A process

In a theoretical PN/A process without influence of NOB, denitrifying bacteria or dissimilatory nitrate reduction to ammonium process, the nitrate production and total nitrogen removal are only conducted by anammox process, so two theoretical ratio existed: total nitrogen removal to ammonium nitrogen removal (ΔTN/ΔAmmonia) of 0.86 and nitrate production to ammonium nitrogen removal (ΔNitrate/ΔAmmonia) of 0.11 (as formula 1). So these two ratios of ΔTN/ΔAmmonia and ΔNitrate/ΔAmmonia are the indicated parameters to estimate the stability and efficient of deammonification process. If external nitrite was oxidized to nitrate by NOB, nitrate would be excessively produced, which then led to ΔNitrate/ΔAmmonia more than 0.11. On the other hand, if denitrifying bacteria existed in PN/A system, total nitrogen would be excessively removed, which then led to ΔTN/ΔAmmonia more than 0.86. So a well performed PN/A process relied on the well cooperation of AOB and anammox communities.

Then the restoration process was evaluated from this two ratios variations (Fig. 2). The theoretical ratios of ΔTN/ΔAmmonia and ΔNitrate/ΔAmmonia in anammox process (2.00 and 0.26, respectively) are different from those in PN/A process^34^. As influent nitrite was introduced from day 10, ΔTN/ΔAmmonia went up rapidly and fluctuated between 1.2 and 2. Nitrite introduction meant a promotion of anammox process, so both ratios should increase in this system theoretically. The down trend of ΔNitrate/ΔAmmonia was clear during day 9 to day 50, which indicated the gradually suppressing of NOB activity. After day 50 when this system was totally run as a deammonification way, the sudden absence of influent nitrite caused a short deteriorated performance (day 50–54), and then the system adapted and achieved ratios of ΔTN/ΔAmmonia and ΔNitrate/ΔAmmonia close to 0.80 and 0.11 in average.

### Quantification of functional bacteria

**Figure 3:**
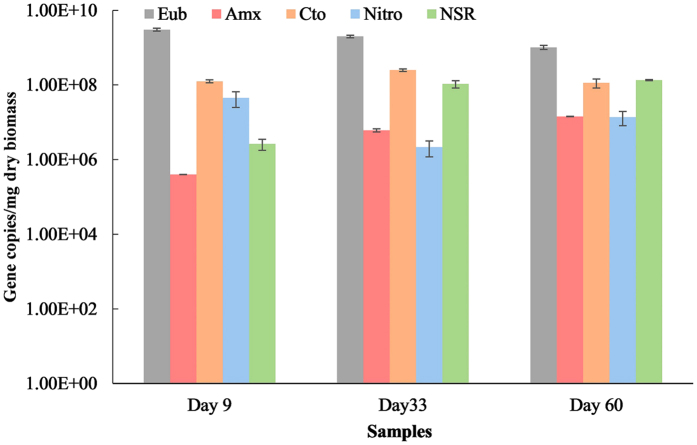
Absolute quantification of functional bacteria.

Quantification of the microorganisms in every stages was performed by quantitative PCR (qPCR) (Fig. 3). After the additional nitrite was introduced, anammox bacteria had a widely growth, with 16S gene increased from 3.98 × 10^5^ copies/mg to 1.43 × 10^7^ copies/mg. Considering the highest nitrogen removal rate (NRR) had reached as high as about 20 kg N/(m^3^·d) on stage II, the anammox bacteria were well protected in the anaerobic compartments of the granular sludge, even under limited aeration environment. The 16S gene copies of AOB was still maintained in the same order of magnitude.

At the stage one when high substrates were introduced, 16S gene copy numbers of *Nitrobacter* declined from 4.52 × 10^7^ copies/mg to 2.17 × 10^6^ copies/mg, and then maintained in the same order of magnitude after the condition of no nitrite addition and ammonium decreasing. But the copy numbers of *Nitrospira* increased before Stage II from 2.63 × 10^6^ copies/mg to 1.06 × 10^8^ copies/mg, and then maintained in the same order of magnitude till Stage III.

## Discussion

### Effect of dissolved oxygen

The one-stage PN/A process has been achieved successfully through different kinks of control strategies. The key is regulating the synergistic relationships between AOB and anammox bacteria, and suppressing NOB growth at the same time. DO concentration is one of the most important parameters in operating PN/A process^26^. The strategy of low DO combined with nitrite introduction, could stimulate the anammox activity that assisted to suppress NOB through competing nitrite^35^. Anammox bacteria is able to compete nitrite with NOB, so it is effective to keep low level of redundant nitrite in the reactor to limit electronic acceptors for NOB^26^. Although the oxygen affinity for AOB (0.04–0.4 mg/L) and for NOB (0.5–1.5 mg/L) variated in different researches, AOB owned the higher oxygen affinity than NOB in the same single habitat in most cases^36^,^37^. By the way, there were also several reports which indicated NOB had higher oxygen affinity than AOB^38^,^39^,^40^. This affinity difference gave AOB the chance to selectively grow up under limited oxygen condition. In one-stage PN/A process, DO levels that AOB preferred were inconsistent with different biomass community, sludge types and reactor configurations, and variated from 0.1 mg/L to about 2 mg/L as below. In granule sludge or membrane bioreactor, DO is able to penetrate into a certain depth of sludge or biofilm to keep aerobic zone for AOB, at the same time, the inside core zone was anaerobic for anammox bacteria growth, so the size of granular sludge and the appropriate DO concentration were often associated to keep the synergistic relationships between AOB and anammox bacteria. To set up DO concentration at 0.6–0.8 mg/L and granule diameter at 2–5 mm, the nitrogen removal efficiency was achieved 81% in an upflow membrane-aerated biofilm reactor^20^. Smaller granule with 3 mm diameter under DO less than 0.5 mg/L also showed the nitrogen removal efficiency of 88%^9^. As to suspended DEMON deammonification system using almost flocculate sludge, DO was strictly control at 0–0.25 mg/L by rapid impulse intermittent aeration^41^. But the single limitation of oxygen supply may be not enough to suppress NOB growth, just as the case in full-scale DEMON plant in Plettenberg^32^. Long operation under DO below 2 mg/L did not solve the nitrate build-up (as the day1–10) in this study as well. It was also reported that the intermittent aeration pattern was more effective to inhibit NOB activity, compared to continuous aeration pattern, without affecting ammonia removal at the same time^42^. In order to suppress NOB activity, we used 1.3 mm diameter of granule, and kept DO level as low as 0.13 mg/L by intermittent aeration. This DO was a very low DO level compared to most studies, which would maintain a favorable environment to suppressing NOB activity.

### Effect of FA and FNA

**Figure 4:**
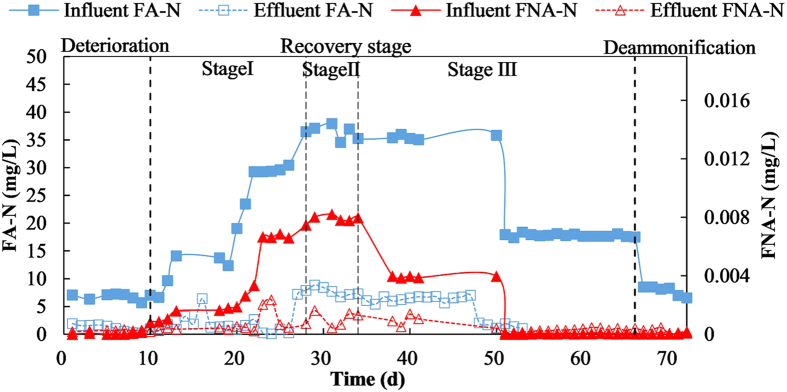
FA-N and FNA-N variations in restoration stages.

High concentration of ammonia or nitrite could inhibit activity of AOB and NOB, in which FA-N and FNA-N are well known as direct inhibitors^43^. Luckily, AOB was able to tolerate about tenfold higher concentration of those two kinds of substrates than NOB. As for FA-N, about 0.1–3 mg/L was most efficient in inhibiting NOB^23^,^44^,^45^. There were 5–40 mg/L of FA-N in influent and not more than 5 mg/L in effluent, both of which would obviously inhibited NOB activity (Fig. 4). As for FNA-N, about 0.02 mg/L was able to inhibited NOB seriously^23^,^46^. Low level of FNA-N existed in influent (<8 μg/L) and effluent (<1 μg/L) because of high pH, so there should be a low inhibition effects on NOB. FA-N and FNA-N also had inhibition effect on anammox bacteria. The inhibiting concentration of FA-N and FNA-N to anammox bacteria were above 50 mg/L and 5 μg/L, respectively^47^. So those substrate levels had little influent on activity of AOB or anammox bacteria. So the inhibition from FA-N combined with limited DO level would be the main factors to inhibit NOB activity^20^.

NOB was always more sensitive to FNA than FA. It was reported that FNA also had a strong biocidal effect on NOB at ppm (mg/L) levels^20^, but this biocidal effect was almost ignorable in our study. And this FNA level need high concentration of nitrite and relative low pH level. Most one-stage PN/A systems were with high ammonia level and relative high pH level, so FA would become an important inhibitor to NOB activity in many studies^30^,^44^,^48^. This was also the reason why NOB was easily suppressed in PN/A process with high influent ammonium (more than 280 mg/L) even under relatively high DO levels or low temperatures^9^,^29^. But 10 mg/L or 23 mg/L FA unexpectedly failed to suppress NOB growth^26^,^49^. For the application of treating low ammonia wastewater, such as municipal wastewater (50–70 mg/L ammonia), the inhibition of FA or FNA on NOB would be tiny. The inhibition of FA also variated along with the biomass morphologies. Compared to flocs, granules have more stronger tolerance to higher FA-N levels such as 5–10 mg/L^50^. It was also reported that the inhibitory threshold of the FA on the PN/A process could reach as high as 77 mg/L^51^. Although FA is the promising inhibited factor to NOB growth, in a particular habit the working inhibition level of FA would adjust according to the actual conditions.

### Evolution of functional bacteria

*Nitrospira* and *Nitrobacter* were two common NOBs existed in wastewater treatment plant, but were unwelcome in PN/A process. In many PN/A systems treating wastewater with high ammonium concentration, it seems that the abundance and activity of NOB can be totally suppressed to undetectable level from the beginning of start-up stage in long-term operation^29^,^30^,^52^. These two NOB species were detected at a large copies numbers in this reactor. So this was the reason why the nitrate build-up appeared.

It was reported that *Nitrospira*-like NOB behaved as K-strategists with high substrate affinity to adapt to low nitrite and oxygen concentrations. On the contrary, *Nitrobactor*-like NOB behaved as r-strategists with low substrate affinity to adapt to high nitrite and oxygen concentrations. So *Nitrospira* often out-completed *Nitrobactor* under limited DO in deammonification reactors^10^. In this research, with the introduced rising nitrite concentration, *Nitrobacter* was inhibited to a certain extent, but *Nitrospira* increased. The phenomenon was consistent with other studies^30^.

After the restoration, even though the gene copy numbers of NOB were still high, the two ratios of ΔTN/ΔAmmonia and ΔNitrate/ΔAmmonia were close to their theoretical values. This suggested that abundant NOB existed but with low activity in the reactor. The inconformity between abundance and activity was also reported in other studies. The AOB abundant did not correlate with the nitritation performance in full-scale petroleum refinery wastewater treatment plant^53^. As the operation temperature decrease, the copies numbers of NOB was stable at around 40 copies/ng DNA while its activity increased from undetectable to significant, which seemed that NOB could hide its activity under inhabitation situations^54^.

It seemed that NOB were persistent in the sludge and could not be easily washed out, especially after NOB community had built up to a certain percentage^55^. In the intermittent aerated-EGSB, NOB in floc sludge was easily washed out for its low settleability. Selective wash-out protocol of NOB by a short SRT has proved to be effective in suspended and completely mixed PN/A system, but was unuseful in biofilm or granule sludge system like in this study. In this study, the floc sludge harbored high abundant AOB (4.72 × 10^9^ copies/mg) than the granular sludge, while NOB had same order of magnitude in floc and granular sludge. Therefore, the undesirable NOB was persistent in granules.

Regardless, the high NOB abundant is always a penitential threat to the PN/A process. Sometime NOB activity may increase again after changing operation pattern even under low NOB abundant^56^. If NOB contamination was serious, the problem of nitrate build-up could be simply solved only by discarding the sludge with more NOB and reinoculation, if enough seeding sludge with anammox bacteria was available conveniently^26^. The nitrate build-up also reappeared after reinoculation^32^. So the best way to suppress NOB is to optimize the operation conditions for dominating AOB in the system at the beginning of the start-up stage to compete NOB growth, and to utilize the multiple inhibitors discussed above will be a promising way. At the stage III, *Nitrobacter*-like or *Nitrospira*-like NOB almost maintained stable which indicated the failed wash-out NOB from granular sludge system, but only with the successful suppress of the NOB activity^30^.

Several NOB inhibitors were already tested in restoration of PN/A system, such as hydrazine, hydroxylamine and organic matter as mentioned in the introduction part. Hydrazine and hydroxylamine are highly toxic intermedia to human, animals, plants or bacteria at low levels, and NaNO_2_ is obviously much safer and more economical to add into the wastewater treatment plant^57^,^58^. As to organic matter, simultaneous partial nitrification, anaerobic ammonium oxidation and denitrification (SNAD) was already successfully developed in many studies^59^,^60^. PN/A process could be also restored by introduction of additional denitrification pathway. But the influent C/N ratio highly affected the SNAD performance, and the autotrophic anammox also easily inhibited by the organic matter^61^. The restoration is more like an emergency processing, not a common operation strategy. And the combination of several inhibition methods and control strategies is recommended.

In conclusion, the deteriorated one-stage PN/A system with nitrate build-up was successfully restored, and the NOB activity was suppressed using elevated substrates level (up to 420 mg/L) under limited DO level (<0.13 mg/L). High FA concentration and low DO took responsibility for this successful restoration. In the restoration stages, anammox bacteria and *Nitrospira*-like NOB had a big growth, while AOB almost stayed stable, while *Nitrobacter*-like NOB decreased. Although the activity of NOB was suppressed of successfully after the restoration, high gene copy numbers of NOB still existed in the system, which was difficult to wash-out without manual sludge discharge.

## Methods

### Reactor configuration and operation

**Figure 5:**
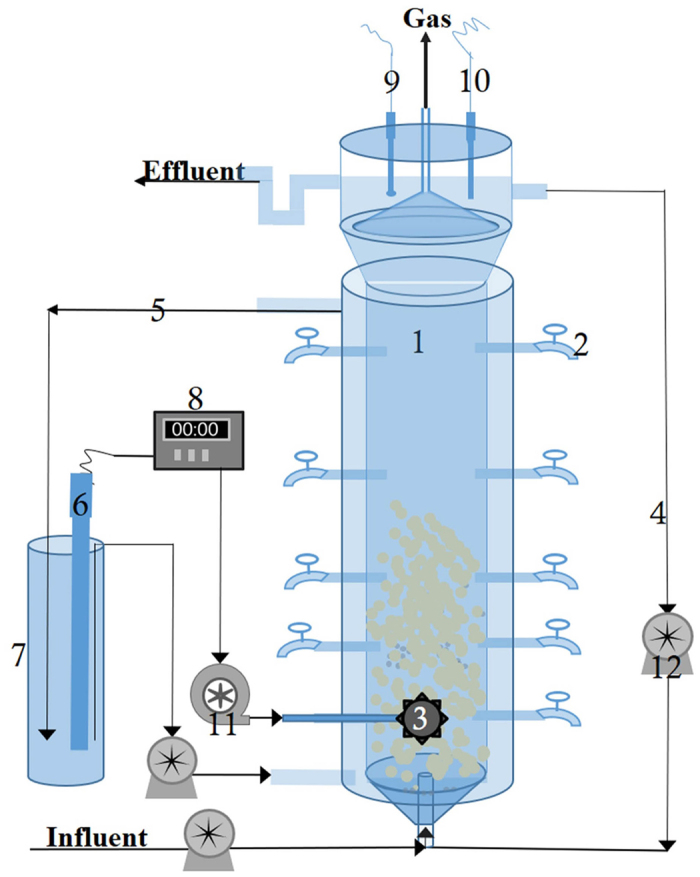
The schematic of the intermittent aerated EGSB system. 1 The EGSB body; 2 Sample ports; 3 Microporous diffusers; 4 Recirculation; 5 Warm water circulation; 6 Heater; 7 Warm water tank; 8 Temperature controller; 9 DO Probe; 10 ORP probe; 11 Aerator; 12 Peristaltic pump.

A lab-scale EGSB (expanded granular sludge bed) was modified with a set of aeration equipment to culture AOB-anammox granule, which just called intermittent aerated EGSB (Fig. 5). This reactor had a working volume of 0.5 L (height of 0.4 m, diameter of 4 cm), and a total capacity of 1.3 L, which was made of polymethyl methacrylate. A water jacket was used to maintain a suitable temperature of 32 °C. Blackout fabric was covered to avoid light inhibition. The aeration rate was adjusted by gas flowmeter along the monitoring data of DO level. Aeration pump was turned on every 3 min and then hold for 30 s controlled by the time relay. The supernatant above the triphase separator was recirculated at about 300 mL/min resulting an upflow velocity of about 16 m/h. The pH was kept on 7.7 in reactor via addition KHCO_3_ in influent, and the measured ORP varied from 60 to120 mV in this reactor.

### Inoculation and operation

The reactor was inoculated with nitrifying sludge (130 mL) and anammox granular sludge (100 mL), which came from storage floc nitritation sludge and a lab-scale EGSB culturing anammox granule, respectively. But most of the flocs were washed out gradually in the later operation stages. Almost only granular sludge was selectively retained and the MLVSS (Mixed Liquor Volatile Suspended Solids) was 280 g/L. The ammonium, nitrite and inorganic carbon in the synthetic wastewater were supplied in the forms of ammonium sulfate, sodium nitrite and potassium bicarbonate, respectively. Trace element solution and vitamin solution were prepared according to previous formulation (Table S1). By excess aeration, the phenomenon of nitrate build-up in effluent appeared, which caused the bad nitrogen removal performance.

The restoration process was implemented as below. Under limited oxygen condition (<0.13 mg/L), added external nitrite into the influent and elevated concentrations of both ammonia and nitrite (<420 mg/L) to introduce the substance inhibition to NOB. After high substance concentrations inhibition (420 mg/L), substance concentrations reduced to the original levels and then examined the autotrophic nitrogen removal performance after restoration process.

### Analytical methods

The concentrations of ammonium (NH_4_^+^), nitrite (NO_2_^−^-N) and nitrate (NO_3_^−^-N) were measured according to the standard methods by a spectrophotometer (T6-1650F, Persee, Beijing, China)^62^. DO, ORP and pH were measured and recorded as the manufacturer’s instructions (Multi 3430, WTW, Munich, Germany). Concentration of FA and FNA was calculated as reported, in which the temperature value was taken as 32 °C, and the pH value was taken as 7.9 and 7.7 in influent and effluent, respectively^63^.

### DNA extraction and quantification

Equal granular biomass from different sample ports were mixed and harvested on day 9, 33 and 60, and then stored immediately at −20 °C after washed by PBS buffer solution. Genome DNA was extracted from samples with particular dry weight using FastDNA SPIN Kit (MP Biomedicals, CA, USA) and evaluated by spectroscopic methods. Primer pairs of Eub 341F/534R, AMX 808F/1040R, CTO 189F/654R, Nitro 1198F/1423R and NSR 1113F/11264R were amplified to produce the standard for qPCR. The detailed information of every used prime pair listed below (Table 1). The PCR mixture and program was set as the manual of Taq polymerase (Taq HIFI, Transgen, beijin, China) on thermal cyclers (T-Gradient Trio PCR, Biometra, Germany). The PCR products were electrophoresed with 1.5% agarose gel on electrophoresis system.

Every successful PCR product was directly ligated into a pUCm-T vector and transformed into DH5α chemically competent cells using pUCm-T Vector Cloning Kit (Sangon Biotech, Shanghai, China). The white colonies including the inserts were pick out and the plasmids were extracted with Easy Pure^TM^ Plasmid MiniPrep Kit (Transgen, beijin, China) and then accurately evaluated by NanoDrop 8000 spectrophotometer (NanoDrop 2000, Thermo Fisher Scientific, MA, USA) as the standard substances for absolute quantification of qPCR.

The copy numbers of the 16S rDNA genes of total bacteria, anammox bacteria, AOB and NOB were determined with absolute quantitative PCR (qPCR) using SGExcel FastSYBR Mixture (With ROX) Kit (Sangong Biotech, Shanghai, China). To construct standard curves for every primer pair, every quantified recombinant plasmid mentioned above was serially diluted in 10-fold steps as standard template. For all qPCR amplifications, a 25 μL reaction containing 12.5 uL of FastSYBR Mixture, 0.5 uL of each primer, 1 μL template DNA, 10.5 μL ddH_2_O was used. Each reaction performed in triplicate. The qPCR protocol was as follows: 94 °C for 20s, 40 cycles of 94 °C for 30s, annealing for 45s and extension at 72 °C for 45s, followed by a melting curve step for the examining specificity of the amplification. The cycle thresholds were determined automatically by SDS software of ABI 7500 Real-Time PCR System (Applied Biosystems, CA). Relative copy numbers among target organism were calculated by excel software.

## Additional Information

**How to cite this article**: Wang, X. and Gao, D. *In-situ* restoration of one-stage partial nitritation-anammox process deteriorated by nitrate build-up via elevated substrate levels. *Sci. Rep.* **6**, 37500; doi: 10.1038/srep37500 (2016).

**Publisher's note:** Springer Nature remains neutral with regard to jurisdictional claims in published maps and institutional affiliations.

## Supplementary Material

Supplementary Information

## Figures and Tables

**Table 1:** Prime pairs used in this study.

Primer	Sequence (5′ to 3′)	Length	Annealing Temperature	Target
Eub341F	cctacgggaggcagcag	17	58.9	Eubacteria
Eub534R	attaccgcggctgctggc	18		
AMX-808F	arcygtaaacgatgggcactaa	22	55.7	Anammox bacteria
AMX-1040R	cagccatgcaacacctgtrata	22		
CTO189F	ggagvaaagyaggggatcg	19	55.5	*Nitrosospira sp. Nitrosomonas sp.*
CTO654R	ctagcyttgtagtttcaaacgc	22		
Nitro-1198F	acccctagcaaatctcaaaaaaccg	25	59.2	*Nitrobacter sp.*
Nitro-1423R	cttcaccccagtcgctgacc	20		
NSR 1113F	cctgctttcagttgctaccg	20	56.8	*Nitrospira sp.*
NSR1264R	gtttgcagcgctttgtaccg	20		
