# Supplementary material for: In-situ restoration of one-stage partial nitritation-anammox process deteriorated by nitrate build-up via elevated substrate levels
Source: Sci Rep. 2016 Nov 24;6:37500. doi: 10.1038/srep37500 (PMC5121595; doi:10.1038/srep37500)
Supplement: Supplementary Information [file srep37500-s1.pdf]

In-situ restoration of one-stage partial nitritation-anammox process deteriorated by nitrate build-up via elevated substrate levels

Xiaolong Wang, Dawen Gao*

State Key Laboratory of Urban Water Resource and Environment, Harbin Institute of Technology, Harbin 150090, China;

*Corresponding author, E-mail: gaodw@hit.edu.cn; Tel: 86-451-86289185; Fax: 86-451-86289185.

Table S1 The constitute of the synthetic waste water.

Substrate	Dosage /L
$CaCl_2$	7 g
KH_2PO_4	1.25 g
$MgSO_4$	10 g
$(NH_4)_2SO_4$	X g
$NaNO_2$	Y g
$KHCO_3$	2.4 g
Trace solution	1 mL
Vitamin solution	1 mL

Trace solution	Dosage /L
$ZnSO_4·7H_2O$	430 mg
$CuSO_4·5H_2O$	250 mg
$MnCl_2·4H_2O$	990 mg
$NiCl·6H_2O$	190 mg
$CoCl_2·6H_2O$	240 mg
H_3BO_4	14 mg
$NaSeO_4$·10H2O	210 mg
$NaMoO_4·2H_2O$	220 mg
EDTA-2Na	15 g
$NaWO_4·2H_2O$	50 mg

Vitamin solution	Dosage /L
Vitamin B6 (pyridoxine) hydrochloride	10 mg
Vitamin B1 (thiamine) hydrochloride	5 mg
Vitamin B2 (riboflavin)	5 mg
Nicotinic acid (niacin)	5 mg
Pantothenic acid (DL Ca-pantothenate)	5 mg
PABA (p-aminobenzoic acid)	5 mg
Lipoic (thioctic) acid	5 mg
Biotin	2 mg
Folic acid	2 mg
B12 (crystalline)	0.1 mg

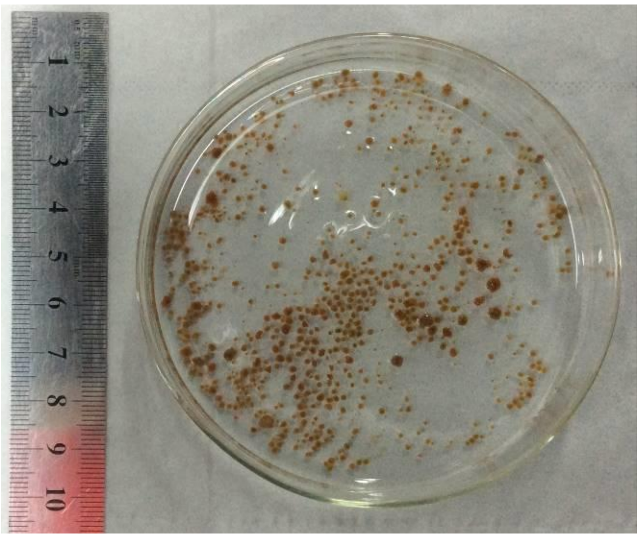

Figure S1 The figure of biomass
